# Inhibitory effect of the multi-target TKI, anlotinib, in 5-FU resistant colorectal cancer HCT-8/15 cells: down regulation of drug resistance-associated protein expression

**DOI:** 10.3389/fonc.2026.1792045

**Published:** 2026-04-10

**Authors:** Juan Liu, Haolin Sun, Xixi Zheng, Nina Ma, Xiaoling Liu, Ruizhen Cao, Bangwei Cao, Bing Liu

**Affiliations:** 1 Cancer Center, Beijing Friendship Hospital, Capital Medical University, Beijing, China; 2 Shanxi Cancer Hospital Affiliated to Shanxi Medical University, Taiyuan, Shanxi, China; 3 Department of Gastroenterology, Ordos Central Hospital, National Clinical Research Center for Digestive Disease-Ordos sub-center Ordos, Inner Mongolia, China; 4 Emergency Department, Beijing Friendship Hospital, Capital Medical University, Beijing, China

**Keywords:** 5-fluorouracil, anlotinib, colorectal cancer, drug resistance, multi-target TKI

## Abstract

**Purpose:**

Colorectal cancer is the most prevalent gastrointestinal malignancy. Treatment usually includes 5-fluorouracil (5-FU), oxaliplatin, and irinotecan, with 5-FU usually being the first choice. 5-FU treatment failure occurs when cancer cells acquire resistance. Therefore, it is crucial to identify compounds effective against 5-FU-resistant tumors. Herein, we determined the efficacy and mechanism of anlotinib in 5-FU-resistant colon cancer cells.

**Materials and methods:**

Human colon cancer cells (HCT-8/5-FU and HCT-15/5-FU) resistant to 5-FU were subjected to treatment with anlotinib, 5-FU, or both. Cell proliferation was assessed via MTS and clone formation assays. Cell cycle progression was studied using flow cytometry. Through immunoblotting, we evaluated changes in the protein levels of p-AKT and multidrug resistance 1.

**Results:**

MTS assays indicated that HCT-8/5-FU and HCT-15/5-FU cells were sensitive to anlotinib and resistant to 5-FU. At 48 h, HCT-8/5-FU had an IC50 of 2246.5 ± 204.5 μM, while HCT-15/5-FU had an IC50 of 18.49 ± 3.23 mM for 5-FU. The IC50 of anlotinib for HCT-8/5-FU cells was 53.69 ± 8.10μM at 24 h and 17.39 ± 1.98μM at 48 h. The IC50 values for HCT-15/5-FU at 24 and 48 h were 55.03 ± 3.44μM and 8.83 ± 3.02μM, respectively. Anlotinib enhanced 5-FU sensitivity in resistant cells, with low concentrations (IC10) considerably enhancing the antiproliferative effects of 5-FU. Further, anlotinib significantly increased the number of cells in the G0-G1 phase dose-dependently, while the proportion of cells entering S phase decreased. MDR1 and AKT expression decreased with increasing anlotinib concentration.

**Conclusion:**

Anlotinib suppressed the proliferation of 5-FU-resistant colon cancer cells by preventing entry into S phase, thus sensitizing cells to 5-FU. Moreover, anlotinib may reverse the effect of 5-FU on drug-resistant cells by down-regulating the expression of multidrug-resistant proteins, in which the AKT signaling pathway may play an important role.

## Graphical Abstract

**Figure:**
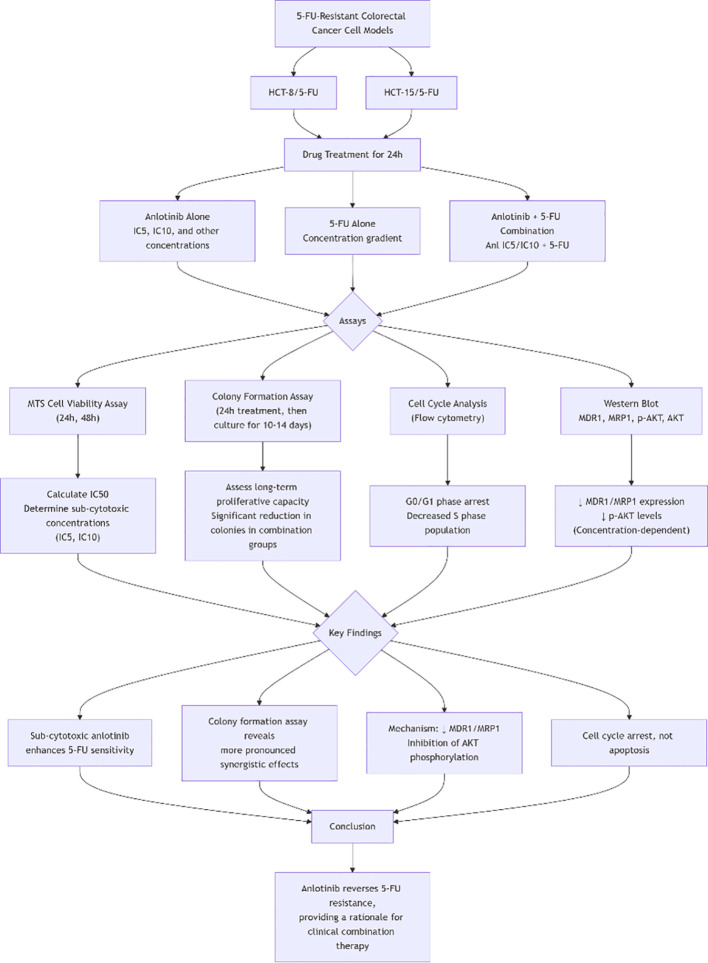
Research flowchart.

## Introduction

Colorectal cancer is the most common gastrointestinal malignancy, ranking second and fourth in terms of mortality and morbidity, respectively, and thus posing a heavy burden on public health (1). Chemotherapy remains the standard of care, even though other therapeutic approaches, including targeted therapy, immunotherapy, and combination therapy, are available (2). The three most widely used chemotherapy drugs for colorectal cancer are 5-fluorouracil (5-FU), oxaliplatin, and irinotecan. The first-line treatment is 5-FU combined with oxaliplatin or irinotecan. However, cancer cells quickly develop resistance to 5-FU, which leads to treatment failure. Clinically, resistance to 5−FU typically emerges after several cycles of therapy (e.g., within 3–6 months of treatment initiation) and is associated with cumulative dose administered, although individual patient factors also contribute to variability. Current strategies to overcome or prevent 5−FU resistance, such as combination with other chemotherapeutic agents, targeted therapies, or modified treatment schedules, have shown limited efficacy, and no established approach reliably averts resistance in clinical practice. Therefore, the identification of therapeutics effective against 5−FU−resistant colon cancer cells is crucial. Multidrug resistance (MDR) is the most common cause of chemotherapy failure, with ATP−binding cassette (ABC) drug transporters playing an important role in tumor MDR. The three most important ABC transporter family members are ABCB1 (P−gp/MDR1), ABCC1 (MRP1), and ABCG2 (BCRP). Moreover, various studies have shown that MDR1 overexpression is the main cause of MDR.

Anlotinib is a novel oral tyrosine kinase inhibitor with several targets. It strongly inhibits the vascular endothelial growth factor receptor (VEGFR2/3), fibroblast growth factor receptor (FGFR1–4), and platelet−derived growth factor receptor (PDGFR). Kinases PDGFR and c−kit are two receptor tyrosine kinases linked to angiogenesis and tumor growth, for which antitumor properties have also been reported (3–5). AKT is the most commonly studied signaling pathway in research on anlotinib in cancer. Anlotinib has been approved for treating advanced non−small−cell lung cancer as well as soft tissue sarcoma. Its efficacy and safety against metastatic colorectal cancer (mCRC) were discussed at the 2018 Chinese Society of Clinical Oncology (CSCO) meeting. Anlotinib monotherapy had an objective response rate (ORR; primary endpoint) of 6.45% and a disease control rate (DCR) of 87.1%. The ALTER0703 trial (NCT02332499) confirmed that anlotinib significantly prolongs progression−free survival (PFS) as a third−line or subsequent treatment in Chinese patients with mCRC (6). In 2022, results from the NCT04080843 trial where anlotinib was combined with chemotherapy as first−line treatment of mCRC with wild−type RAS/BRAF showed a considerable improvement in ORR, DCR, PFS, and duration of response (DOR). Many trials evaluating anlotinib against advanced colorectal cancer are currently ongoing. Although anlotinib efficacy has been confirmed in clinical trials, its specific mechanism of action against colorectal cancer cells has been poorly studied. As a TKI, anlotinib is thought to exert its effects by competitively binding to ATP−binding sites, thereby attenuating downstream signaling pathways associated with tumor proliferation, invasion, migration, and angiogenesis.

Anlotinib blocks numerous signaling pathways, and little is known regarding its effects in drug−resistant colon cancer cells. In this study, we combined pharmacological assays with morphological analysis (e.g., observing changes in cell morphology following anlotinib treatment) to investigate the effects of anlotinib on 5−FU−resistant colorectal cancer cells and its underlying mechanisms. Materials and methods included cell culture, establishment of 5-FU-resistant cell lines, MTT assays for cell viability, morphological observation under light microscopy, flow cytometry for apoptosis analysis, and western blotting to assess MDR1 and AKT pathway protein expression. Specifically, we sought to determine whether anlotinib is effective against 5−FU−resistant colorectal cancer cells and whether it might reverse resistance by suppressing the MDR1 and AKT pathways. Elucidating this mechanism could provide a novel therapeutic strategy for patients with acquired 5−FU resistance and inform future clinical trials exploring anlotinib in combination with chemotherapy for resistant colorectal cancer.

## Methods

### Cell culture and reagents

HCT-8 cells were purchased from ATCC and cultured in Dulbecco’s Modified Eagle’s Medium (DMEM, Gibco, USA) supplemented with 10% fetal bovine serum (FBS, Gibco, USA). The cells were maintained at 37°C in a humidified atmosphere containing 5% CO_2_. HCT-15 and 5-fluorouracil (5-FU)-resistant HCT-15 cells (HCT-15/5-FU) were obtained from Shanghai Yihe Biotechnology (China). 5-FU-resistant HCT-8 cells (HCT-8/5-FU) were obtained from Beijing Being Biotechnology (China). The resistant cell lines were maintained in medium containing 5-FU at a final concentration of 1 μg/mL for HCT-8/5-FU cells and 3.2 μg/mL for HCT-15/5-FU cells to sustain their resistant phenotype. Anlotinib was purchased from Chia Tai Tianqing Pharmaceutical Group Co., Ltd. (Nanjing, China). MTS reagent was purchased from Promega Corporation (Madison, WI, USA). Propidium iodide was purchased from Sigma-Aldrich (Saint Louis, MO, USA).

### MTS assay

Drug cytotoxicity and resistance were assessed using the MTS test. Briefly, 5,000 cells at the logarithmic growth phase were seeded in 96-well plates and were grown for 24h at 37°C. Cells were then treated with various 5-FU or anlotinib doses for 24h and 48h, followed by a 2h incubation period after the addition of 20µL MTS reagent (CellTiter 96^®^ AQueous One Solution Cell Proliferation Assay, Promega Corporation, USA) to each well. Absorbance at 490 nm was obtained using a Bio-Rad Microplate Reader Model 680(Spectramax M3; Molecular Devices, USA). The assays were conducted in triplicate.

### Colony formation assays

One thousand logarithmic growth cells were seeded in 6-well plates, followed by incubation for 24 h. Thereafter, cells were re-incubated and cultivated for a further 24h in medium with drugs at various doses. Following a further 12 days of cultivation in drug-free medium, the cells were fixed in 10% formaldehyde, stained with Giemsa, rinsed with phosphate-buffered saline (PBS), and imaged. This was performed in triplicate.

### Cell cycle analysis

To examine the effect of anlotinib on 5-FU-resistant colon cell lines, we determined cell cycle distribution in HCT-8/5-FU and HCT-15/5-FU. HCT-8/5-FU and HCT-15/5-FU were seeded in 6-well plates. Cells were separated into four groups and supplied with different amounts of anlotinib for 24h once they reached 70-80% confluence. Prior to trypsinization, cells were centrifuged at 1,000 rpm for 3min, followed by two rounds of washing in ice-cold PBS. The cells were then fixed in 3mL of 70% cold ethanol at 4°C overnight. The fixed cells were centrifuged, washed with PBS, and treated with 250μL propidium iodide at room temperature for 15min. Finally, cell cycle distribution was analyzed using a FACScan (BD FACSCalibur™, USA).

### Immunoblotting

Immunoblotting was used to assess phosphorylation and protein levels. HCT-8/Fu and HCT-15/Fu cells were seeded in 6-well plates and grown to 70-80% confluence. Following respective treatments (different drug concentrations for 24h), cells were lysed in HEPES 50µM, NaCl 150µM, EDTA 1mM, 1% Triton, and 10% glycerol supplemented with protease inhibitors. The lysates were centrifuged at 4°C at 12,000 rpm for 15min.A BCA protein assay kit (Merck, Darmstadt, Germany) was used to measure total protein concentration. After separation on a 10% SDS-PAGE gel, proteins were transferred to a PVDF membrane (Millipore) and blocked in 5% non-fat milk. Membranes were incubated with primary antibodies overnight at 4°C and secondary antibodies for 2h at room temperature. TBST buffer was used to wash off the unbound antibodies. Protein bands were visualized on an ECL plus system (Beyotime). Image J software(USA) was used for image analysis. The following specific primary antibodies were used for western blot analysis: MRP1/ABCC1 rabbit antibody (1:1000, 72202S, CST); MDR1/ABCB1 rabbit antibody(1:1000, 13342, CST); GAPDH mouse monoclonal antibody (1:5000, Cat. No:60004-1-Ig, Proteintech); p-AKT rabbit monoclonal antibody (1:1000, 4060S, CST); and AKT rabbit polyclonal antibody (1:1000, Cat. No:10176-2-AP, Proteintech).

### Statistical analysis

GraphPad Prism 5.0 software was used for statistical analysis. Data are presented as the mean ± SD. To evaluate statistical significance, we employed Shapiro-Wilk tests for normality of distribution and Barlett tests for homogeneity of variance. For data with normal distribution and homogenous variance, we employed one-way analysis of variance with Tukey *post-hoc* test for multiple comparisons. Nemenyi test were used for multiple comparisons if not satisfied using Kruskal-Wallis tests. Differences were deemed significant at a p-value of 0.05.

## Results

### Inhibitory effects of anlotinib and 5-FU on colon cancer cell proliferation

**Figure 1:**
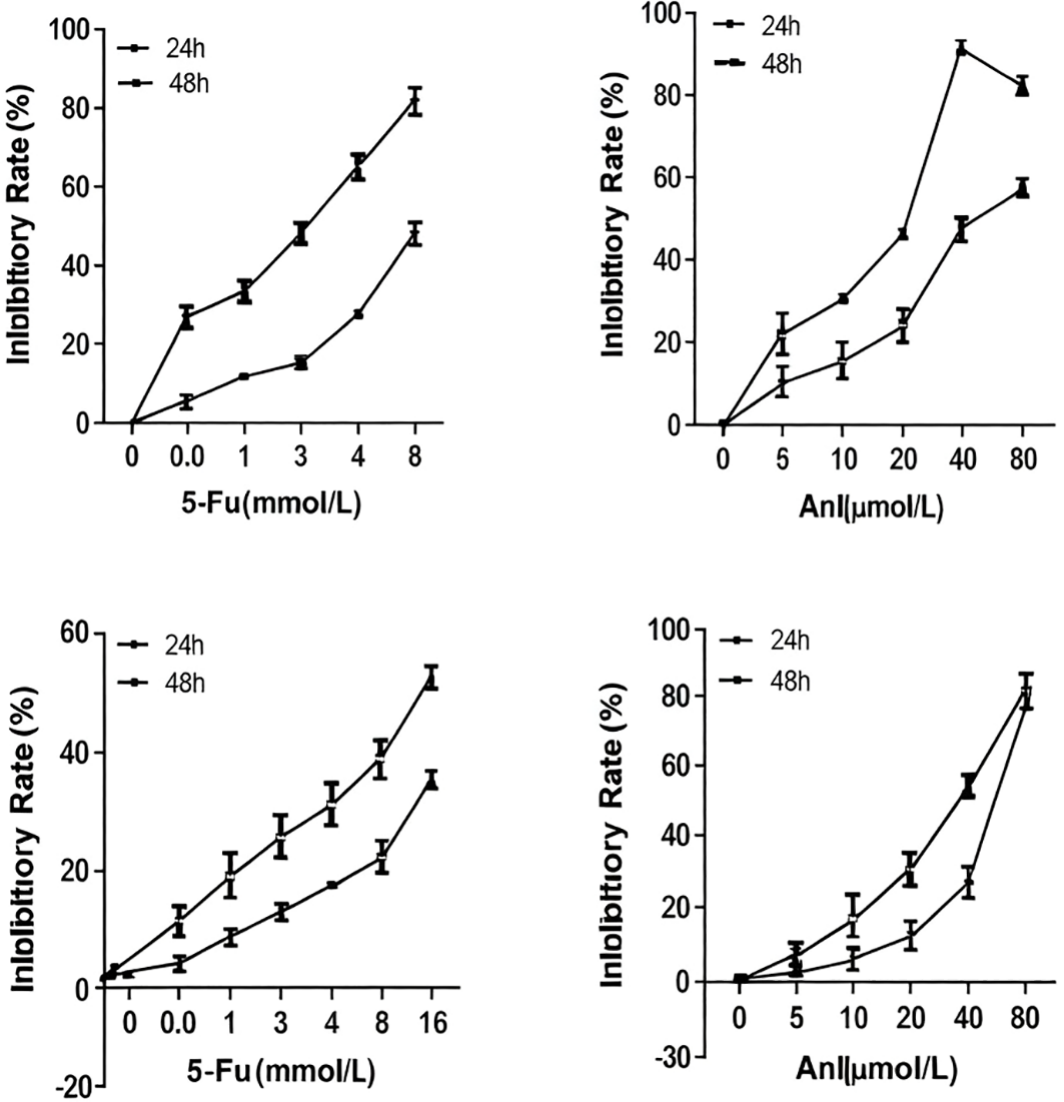
Effect of anlotinib and 5-fluorouracil (5-FU) on colon cancer cell proliferation. **(A)** HCT-8/5-FU cells treated with 5-FU for 24 h and 48 h. IC50 (48 h) = 2246.5 ± 204.5 μM. **(B)** HCT-8/5-FU cells were treated with anlotinib for 24 h and 48 h. IC50 (24 h) = 53.69 ± 8.10 μM; IC50 (48 h) = 17.39 ± 1.98 μM. **(C)** HCT-15/5-FU cells treated with 5-FU for 24h and 48h. IC50 (48 h) = 18.49 ± 3.23 mM. **(D)** HCT-15/5-FU cells treated with anlotinib for 24 h and 48 h. IC50 (24 h) = 55.03 ± 3.44 μM; IC50 (48 h) = 28.83 ± 3.02 µM. Data are presented as the mean ± SD obtained from three independent experiments.

We exposed HCT-8/5-FU and HCT-15/5-FU cells to various concentrations of anlotinib and 5-FU for 24h and 48h in order to examine the impact on cell growth. In both lines, anlotinib and 5-FU inhibited proliferation, which was dependent on time and dose (**Figure 1**). At 48h, the half-maximal inhibitory concentration (IC50) of 5-FU was 2246.5 ± 204.5 μM in HCT-8/5-FU cells and 18.49 ± 3.23 mM in HCT-15/5-FU cells. IC50 values could not be obtained at 24h. To assess the inhibitory activity of anlotinib, we employed a range of concentrations. The observed effects depended on both time and dose. The IC50 for anlotinib in HCT-8/5-FU cells was 53.69 ± 8.10 μM at 24 h and 17.39 ± 1.98 μM at 48 h. At 24h, the IC50 in HCT-15/5-FU cells was 55.03 ± 3.44 μM, compared to 8.83 ± 3.02 μM at 48h.

### Anlotinib promotes 5-FU sensitivity in resistant colon cancer cells

**Figure 2:**
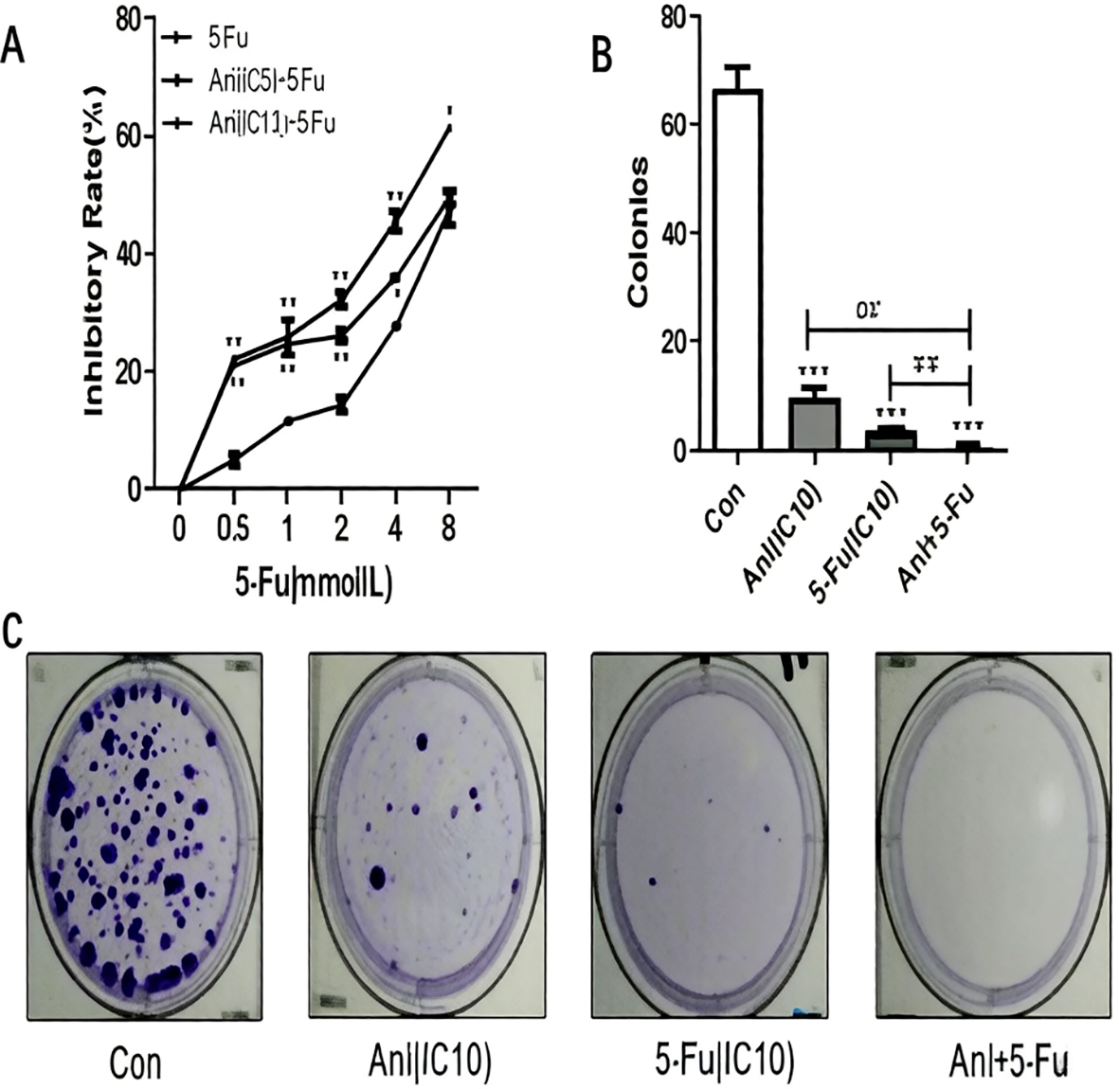
Anlotinib reverses of 5-FU resistance in HCT-8/5-FU colon cancer cells. **(A)** HCT-8/5-FU cells were treated with anlotinib (at IC5 and IC10) and 5-FU at specified concentrations for 24 h. Cell viability was evaluated using the MTS assay. Data are presented as the mean ± SD. Doses below the IC10 are typically regarded as safe. In order to avoid cytotoxicity attributed to high doses rather than sensitization, we used IC10 in this experiment. **(B)** Colonies formed in three independent experiments were calculated from **(C)**. **(C)** Representative photos of HCT-8/5-FU colonies in the control, 6 µM anlotinib (IC10), 2 mM 5-FU (IC10), and combined treatment group. Following treatment with the indicated reagents, the number of colonies formed per dish was assessed. *p<0.05 represents the comparison between the experimental and the control group; #p<0.05 represents the comparison between the combination and single drug groups. Data represent three independent experiments.

**Figure 3:**
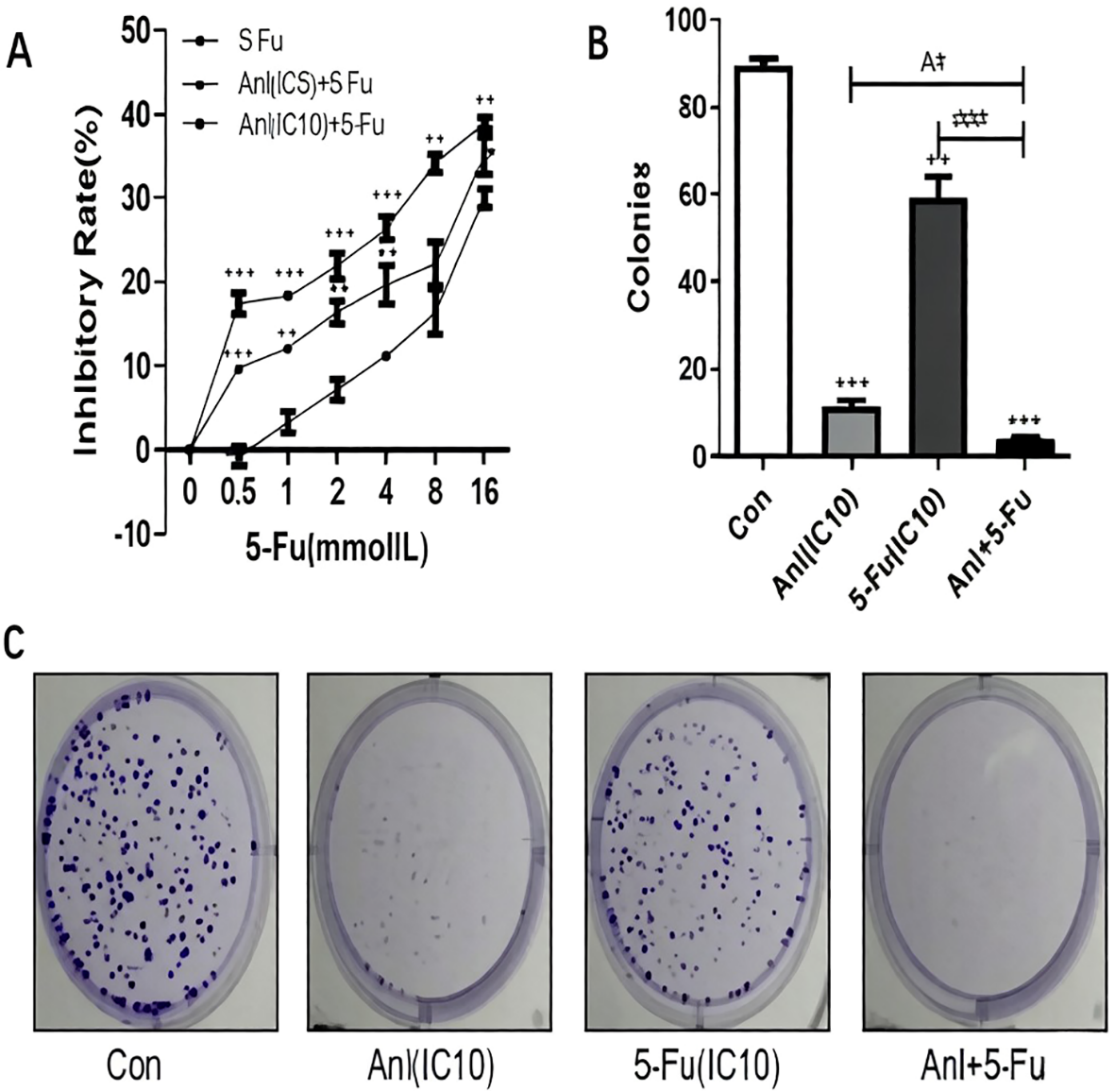
Anlotinib reverses drug resistance in HCT-15/5-FU colon cancer cells. **(A)** HCT-15/5-FU cells treated with anlotinib (IC5 and IC10) together with indicated 5-FU concentrations for 24h. We used MTS assays to determine cell viability. Data are presented as the mean ± SD of three independent experiments. **(B)** Number of colonies formed under the indicated treatment was calculated from **(C)**. **(C)** Representative photos of HCT-15/5-FU colonies under control, 6 μM anlotinib (IC10), 2 mM 5-FU (IC10), and combination treatment. Colonies per dish after indicated treatment. Three experiments were conducted independently. *p<0.05 represents the comparison between the experimental and control groups; #p<0.05 represents the comparison between the drug combination group and single drug groups. Data represent three independent experiments.

We treated HCT-8/5-FU and HCT-15/5-FU cells with anlotinib at a non-lethal dose along with increasing concentrations of 5-FU for 24 h, in order to determine whether anlotinib could sensitize resistant cells to 5-FU. This treatment duration was selected based on the time-dependent cytotoxicity profile of anlotinib (**Figure 1**), as a 48 h exposure exhibited stronger single-agent toxicity that might potentially mask synergistic interactions. In resistant cell lines, doses below the IC10 are typically regarded as safe amounts. We determined the IC5 and IC10 doses of anlotinib in HCT-8/5-FU (3.15 ± 2.30 µM and 6.27 ± 3.74 µM, respectively) and HCT-15/5-FU cells (12.01 ± 3.16 µM and 17.09 ± 3.95 µM, respectively). These sub−cytotoxic concentrations were then used to evaluate the sensitizing effect of anlotinib in 5−FU−resistant cell lines. Notably, the proliferation of HCT-8/5-FU and HCT-15/5-FU cells was synergistically inhibited by the combination of anlotinib and 5-FU. Compared to either single drug alone, the combination induced a significantly higher inhibition rate in MTS assays. Colony formation assays confirmed this synergistic impact to an even greater extent. While anlotinib at its IC10 concentration showed only minimal acute cytotoxicity in the MTS assay, it markedly enhanced the suppressive effect of 5-FU on long−term colony formation, resulting in substantially fewer and smaller colonies in the combination group than in the single−drug groups (**Figure 2** and **3**). This discrepancy highlights the heightened sensitivity of clonogenic assays in detecting durable impairments in proliferative capacity, even after transient exposure to sub−cytotoxic drug concentrations. It is worth noting that experiments using anlotinib at its IC50 concentration in combination with 5-FU were not performed in the current study; therefore, whether higher-dose combinations would produce enhanced or qualitatively different effects remains to be investigated in future studies.

### Effects of anlotinib on cell cycle progression in drug-resistant colorectal cancer cells

**Figure 4:**
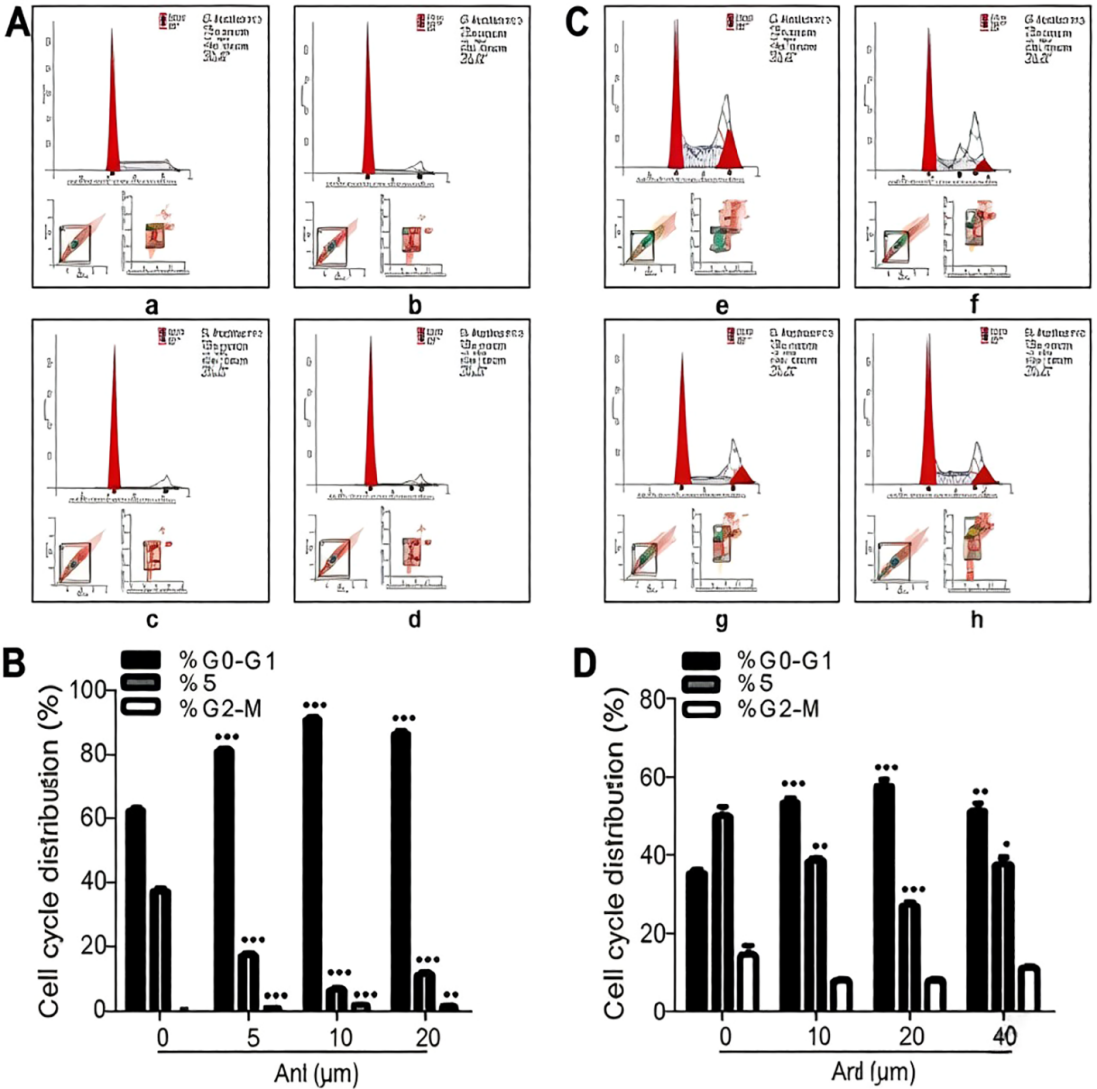
Effects of anlotinib on the cell cycle. **(A, B)** The effect of different anlotinib concentrations (0, 5, 10, 20 µM, in **(a–d)** respectively) on HCT-8/5-FU cells. **(C, D)** HCT-15/5-FU cells assayed as in **(A, B)**, **(e–h)**. Anlotinib reduced the number of cells in the S phase while increasing those in the G0/G1 phase (p<0.05). Concentrations of 20 µM and 10 µM had the greatest effect in HCT-15/5-FU and HCT-8/5-FU cells, respectively. Data are presented as the mean ± SD of three experiments. *p<0.05 represents the comparison between the experimental and control groups. Anlotinib suppressed the viability of HCT-8/FU cells even at 5 µM, while no effect was observed in HCT-15/FU cells. Therefore, 5 µM was not included as a treatment for HCT-15/FU cells. Meanwhile, 80 µM had the strongest cytotoxic effect. To avoid excess cell death, this dose was not selected.

We explored the effects of anlotinib on cell cycle progression in light of its anti-proliferative properties. We treated HCT-15/5-FU cells with 10 µM, 20 µM, and 40 µM doses for cell cycle analysis, as no suppression of proliferation had been observed at 5 µM based on the MTS results. Anlotinib increased the percentage of cells in the G0/G1 phase, with fewer cells entering S phase (**Figure 4**). This effect was most pronounced at 20 µM in HCT-15/5-FU cells and at 10 µM in HCT-8/5-FU cells. Apoptosis was not enhanced under any of the treatment conditions.

### Anlotinib inhibits drug resistance-related protein expression and AKT pathway activation

**Figure 5:**
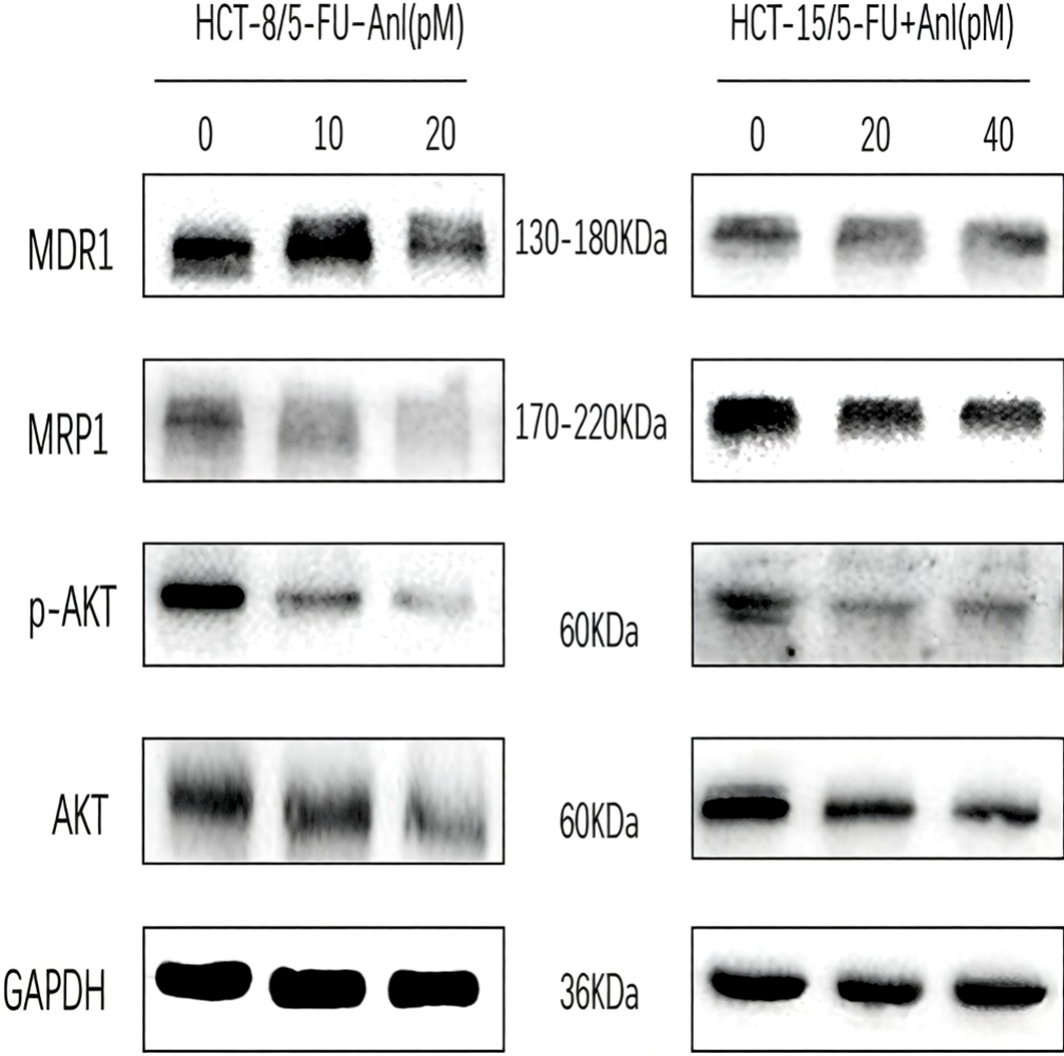
Effect of anlotinib on drug resistance-associated protein expression and AKT signaling in 5-FU-resistant colorectal cancer cells. HCT-8/5-FU and HCT-15/5-FU cells were treated with increasing concentrations of anlotinib (IC5, IC10, and higher doses) for 24 h. Protein levels of MDR1, MRP1, phosphorylated AKT (p-AKT), and total AKT were analyzed by immunoblotting; GAPDH was used as a loading control.

Drug resistance proteins are frequently upregulated in drug-resistant tumor cells. We assessed the expression of resistance-related proteins MRP1 and MDR1 to further interrogate the mechanism of action of anlotinib in colorectal cancer cells. High concentrations were chosen due to the low inhibition rate at lower concentrations, as per MTS results of anlotinib on drug-resistant colon cancer cells. MDR1 and MRP1 were strongly expressed in HCT-8/5-FU and HCT-15/5-FU cells, as determined via immunoblotting. Increasing anlotinib concentration suppressed their expression. We then assessed the levels of total and phosphorylated AKT (**Figure 5**). Increasing anlotinib concentration suppressed p-AKT levels.

Notably, MDR1 and MRP1 were highly expressed in both HCT-8/5-FU and HCT-15/5-FU cells, confirming their drug-resistant phenotype. Treatment with anlotinib suppressed MDR1 and MRP1 expression in a concentration-dependent manner, with significant downregulation observed even at IC5 and IC10 concentrations. Furthermore, anlotinib decreased p-AKT levels in a concentration-dependent fashion, while total AKT expression remained unchanged, suggesting that inhibition of AKT phosphorylation may contribute to the downregulation of drug resistance proteins. These molecular changes provide a mechanistic basis for the enhanced sensitivity to 5-FU observed at sub-cytotoxic anlotinib concentrations in **Figures 2**, **3**.

## Discussion

Colorectal cancer remains one of the leading causes of cancer-related mortality worldwide, and acquired resistance to 5-fluorouracil (5-FU) represents a major clinical obstacle in the management of this disease (7, 8). In the present study, we demonstrated that anlotinib, a multi-target tyrosine kinase inhibitor, effectively sensitizes two 5-FU-resistant colorectal cancer cell lines (HCT-8/5-FU and HCT-15/5-FU) to 5-FU treatment. Notably, this sensitization was achieved at sub-cytotoxic concentrations of anlotinib (IC5 and IC10), suggesting that the observed synergistic effects are not merely attributable to additive cytotoxicity but rather reflect a true pharmacological interaction that re-sensitizes resistant cells to chemotherapy (3).

A key finding of this study is the marked discrepancy between the results of short-term viability assays (MTS) and long-term colony formation assays. While anlotinib at its IC10 concentration exhibited minimal acute cytotoxicity in MTS assays, it profoundly enhanced the inhibitory effect of 5-FU on colony formation, resulting in significantly fewer and smaller colonies in the combination treatment groups (**Figures 2**, **3**). This observation underscores the fundamental difference between these two experimental endpoints: MTS assays measure metabolic activity as a surrogate for short-term viability, whereas colony formation assays assess the capacity for long-term proliferation and self-renewal—properties that are closely linked to tumor recurrence and therapeutic resistance. Our findings suggest that anlotinib, even at doses that do not acutely compromise cell viability, may induce durable growth arrest or senescence in 5-FU-resistant cells, thereby enhancing their susceptibility to chemotherapy. This interpretation is consistent with previous reports demonstrating that tyrosine kinase inhibitors can exert cytostatic effects that manifest more clearly in long-term functional assays (9–11).

The choice of a 24-hour treatment duration for our combination studies warrants further comment. As shown in **Figure 1**, anlotinib exhibited time-dependent cytotoxicity, with 48-hour exposure yielding substantially lower IC50 values. Had we selected a 48-hour treatment for the combination experiments, the stronger single-agent toxicity of anlotinib might have obscured the synergistic interaction with 5-FU, leading to an overestimation of additive effects. By choosing a 24-hour exposure, we established a baseline condition with moderate single-agent activity, allowing for a more sensitive and accurate assessment of synergy. This experimental design consideration is often overlooked but is critical for interpreting drug combination studies, particularly when one agent exhibits time-dependent cytotoxicity.

Mechanistically, we investigated whether anlotinib reverses 5-FU resistance by modulating multidrug resistance (MDR) transporters and the AKT signaling pathway. ATP-binding cassette (ABC) transporters, particularly MDR1 (ABCB1/P-gp) and MRP1 (ABCC1), play a pivotal role in mediating chemotherapy resistance by actively extruding drugs from cancer cells (12–14). In the present study, we observed that anlotinib suppressed MDR1 and MRP1 expression in a concentration-dependent manner in both 5-FU-resistant cell lines (**Figure 5**). This finding is particularly significant, as MDR1 overexpression is widely regarded as the primary driver of multidrug resistance in colorectal cancer (13). Importantly, downregulation of these efflux pumps was observed even at IC5 and IC10 concentrations, consistent with the sensitizing effects demonstrated in **Figure 2** and **3**. By downregulating MDR1 and MRP1, anlotinib may increase intracellular accumulation of 5-FU, thereby restoring its cytotoxic efficacy. Similar mechanisms have been reported for other tyrosine kinase inhibitors, which have been shown to modulate ABC transporter expression and reverse drug resistance in various cancer models.

In addition to its effects on drug transporters, we examined the involvement of the PI3K/AKT signaling pathway, which is frequently hyperactivated in colorectal cancer and contributes to both proliferation and chemoresistance. Our results demonstrated that anlotinib decreased phosphorylated AKT (p-AKT) levels in a concentration-dependent manner, while total AKT expression remained unchanged (**Figure 5**). This observation aligns with previous studies showing that anlotinib suppresses tumor growth by inhibiting AKT signaling in lung cancer, hepatocellular carcinoma, and colorectal cancer (7, 9, 15–18). Notably, the convergence of anlotinib’s effects on both MDR transporters and AKT signaling raises the possibility of a mechanistic link between these two pathways. Emerging evidence suggests that AKT signaling can regulate ABC transporter expression through transcriptional and post-translational mechanisms. Thus, anlotinib-mediated inhibition of AKT phosphorylation may contribute, at least in part, to the downregulation of MDR1 and MRP1 observed in our study. The concentration-dependent nature of these effects, with changes detectable at IC5 and IC10 doses, further supports the relevance of these mechanisms to the synergistic interactions observed at sub-cytotoxic concentrations.

Another important observation from our study is that anlotinib did not significantly enhance apoptosis in 5-FU-resistant cells, despite suppressing proliferation and colony formation. This suggests that the primary mechanism of anlotinib’s sensitizing effect may be cytostatic rather than cytotoxic, potentially involving cell cycle arrest. Indeed, our cell cycle analysis indicated that anlotinib treatment prevented entry into S phase, consistent with its known effects on cell cycle progression in other cancer types. (19, 20) These findings imply that anlotinib may “prime” resistant cells by inducing quiescence or slowing proliferation, thereby rendering them more vulnerable to the anti-metabolite effects of 5-FU. This interpretation is supported by the observation that combination treatment resulted in a greater-than-additive reduction in colony formation, which reflects cumulative effects on proliferative capacity over multiple cell divisions.

Despite the promising findings presented herein, several limitations of this study should be acknowledged. First, experiments using anlotinib at its IC50 concentration in combination with 5-FU were not performed in colony formation assays. While the observed effects at sub-cytotoxic doses (IC5 and IC10) are encouraging and clinically relevant—as low-dose combinations may offer therapeutic benefits with reduced toxicity—a comprehensive dose-matrix analysis including equipotent doses would provide a more complete characterization of the synergistic interaction. Second, formal synergy analysis using established methods such as the Chou-Talalay combination index was not conducted; therefore, the nature of the interaction (synergistic vs. additive) was inferred qualitatively rather than quantitatively. Third, our mechanistic studies were limited to two resistant cell lines and focused primarily on MDR transporters and AKT signaling. It remains possible that other pathways—such as MAPK/ERK, Wnt/β-catenin, or autophagy-related mechanisms—may also contribute to anlotinib’s sensitizing effects, as suggested by previous studies (11, 21–25). Finally, the *in vitro* nature of this study precludes conclusions about the *in vivo* efficacy, pharmacokinetics, or toxicity of anlotinib-5-FU combinations. Future studies using animal xenograft models and, ultimately, clinical specimens will be essential to validate these findings and to assess their translational potential.

From a clinical perspective, our findings have several important implications. Anlotinib has already been approved in China for the treatment of advanced non-small cell lung cancer and soft tissue sarcoma, and its safety profile is well established (3–5). The ALTER0703 trial demonstrated that anlotinib monotherapy prolongs progression-free survival in patients with metastatic colorectal cancer (26), and ongoing trials are evaluating its efficacy in combination with chemotherapy. Our results provide a mechanistic rationale for combining anlotinib with 5-FU-based regimens specifically in patients with acquired 5-FU resistance. Notably, the concentrations at which anlotinib exerted its sensitizing effects in our study (IC5 and IC10) are within the range of clinically achievable plasma concentrations reported in pharmacokinetic studies, supporting the potential translatability of our findings. The fact that these sensitizing effects were achieved at sub-cytotoxic doses suggests that anlotinib-5-FU combinations may offer therapeutic benefits without exacerbating toxicity—a particularly important consideration for patients who have already progressed on multiple lines of therapy.

In conclusion, this study demonstrates that anlotinib, at sub-cytotoxic concentrations, effectively reverses 5-FU resistance in colorectal cancer cells by suppressing MDR1 and MRP1 expression and inhibiting AKT phosphorylation. The striking enhancement of combination effects in colony formation assays, despite minimal acute cytotoxicity, highlights the importance of assessing long-term proliferative capacity when evaluating drug combinations. These findings provide a mechanistic foundation for future preclinical and clinical investigations of anlotinib-5-FU combinations in patients with 5-FU-resistant colorectal cancer, and underscore the potential of repurposing anlotinib as a chemosensitizing agent beyond its approved indications.

## Conclusion

In this study, we demonstrated that anlotinib, at sub-cytotoxic concentrations (IC5 and IC10), effectively reverses 5-FU resistance in colorectal cancer cells. The key findings are as follows: First, anlotinib sensitizes resistant cells to 5-FU in short-term viability assays, and this effect is markedly amplified in long-term colony formation assays, highlighting the importance of assessing proliferative capacity when evaluating drug combinations. Second, the synergistic effects observed at low-dose combinations are mechanistically linked to the downregulation of multidrug resistance transporters MDR1 and MRP1, as well as inhibition of AKT phosphorylation. Third, the choice of a 24-hour treatment duration was critical for unmasking these synergistic interactions, as longer exposure times would have been confounded by anlotinib’s single-agent cytotoxicity.

Notably, anlotinib exerted these sensitizing effects without significantly enhancing apoptosis, suggesting that cytostatic mechanisms—such as cell cycle arrest and suppression of proliferative capacity—may play a dominant role. The concentrations at which anlotinib demonstrated efficacy in our study are within clinically achievable ranges, supporting the translational potential of this approach.

We acknowledge certain limitations, including the absence of IC50-based combination experiments and formal synergy analysis. Future studies employing animal models, a broader range of combination doses, and investigations into additional signaling pathways will be necessary to fully elucidate the therapeutic potential of anlotinib in overcoming 5-FU resistance. Nevertheless, our findings provide a mechanistic rationale for repurposing anlotinib as a chemosensitizing agent in patients with acquired 5-FU-resistant colorectal cancer, and support further evaluation of low-dose anlotinib-5-FU combinations as a potential strategy to improve clinical outcomes while minimizing toxicity.

## Data Availability

The original contributions presented in the study are included in the article/supplementary material. Further inquiries can be directed to the corresponding author.
